# Supplementary material for: Ub-ProT reveals global length and composition of protein ubiquitylation in cells
Source: Nat Commun. 2018 Feb 6;9:524. doi: 10.1038/s41467-018-02869-x (PMC5802829; doi:10.1038/s41467-018-02869-x)
Supplement: Supplementary file 1 — Supplementary Information [file 41467_2018_2869_MOESM1_ESM.pdf]

Supporting Information

Ub-ProT reveals global length and composition of protein ubiquitylation in cells.

Tsuchiya et al.

This PDF file includes:

9 Supplementary Figures
1 Supplementary Table
Supplementary References

Other supplementary items for the manuscript includes the following:

Supplementary Data 1 (Dataset of Supplementary Fig. 2d)
Supplementary Data 2 (Dataset of Fig. 3b)
Supplementary Data 3 (Dataset of Fig. 3d)
Supplementary Data 4 (Dataset of Supplementary Fig. 7a)
Supplementary Data 5 (Dataset of Fig. 4b)
Supplementary Data 6 (Dataset of Fig. 4c)
Supplementary Data 7 (Dataset of Fig. 6c)

Supplementary Figures

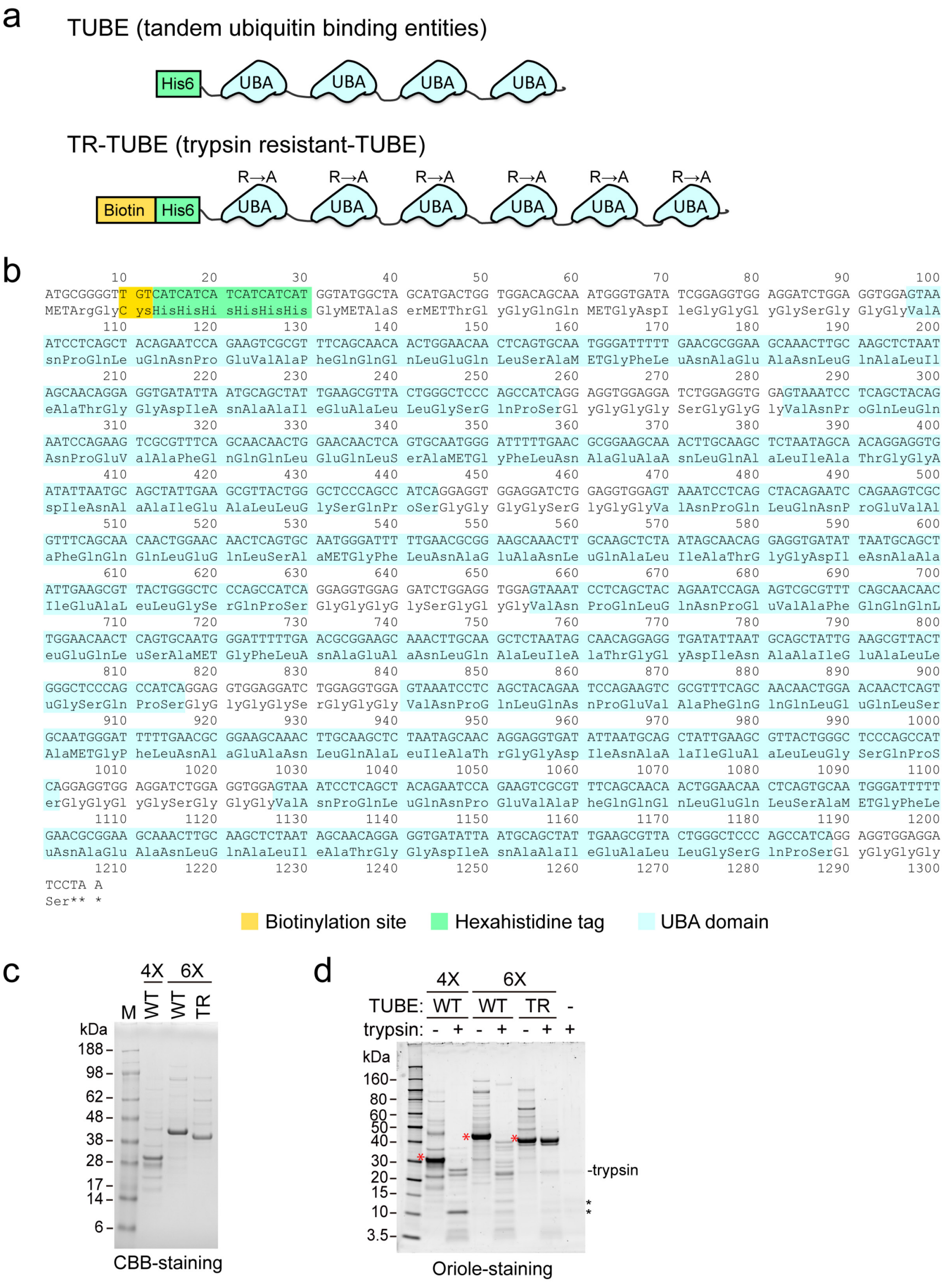

Supplementary Figure 1. Structure, sequence, and purification of trypsin-resistant (TR)-TUBE.

(*a*) Illustrations of the original TUBE construct and TR-TUBE. TUBE, which contains four tandem repeats of the UBA domain of human UBQLN1 with flexible linkers (top), was originally developed by Hjerpe *et al.*[1]. TR-TUBE, used in this study, contains a biotin

(the yellow box), a hexahistidine tag (pale green), and six tandem repeats of the mutated UBA domain (aqua; bottom).

(*b*) DNA and amino-acid sequences of TR-TUBE. The Cys residue for biotinylation, hexahistidine tag, and mutated UBA domains are highlighted as in (*a*).

(*c*) Expression and purification of 4×TUBE, 6×TUBE, and 6×TR-TUBE. Purified proteins were analyzed by SDS-PAGE.

(*d*) Trypsin digestion of TUBEs. Purified TUBEs (1 μg) indicated by red asterisks were incubated overnight at 37°C with trypsin (200 ng). Black asterisks indicate the degradation product of trypsin.

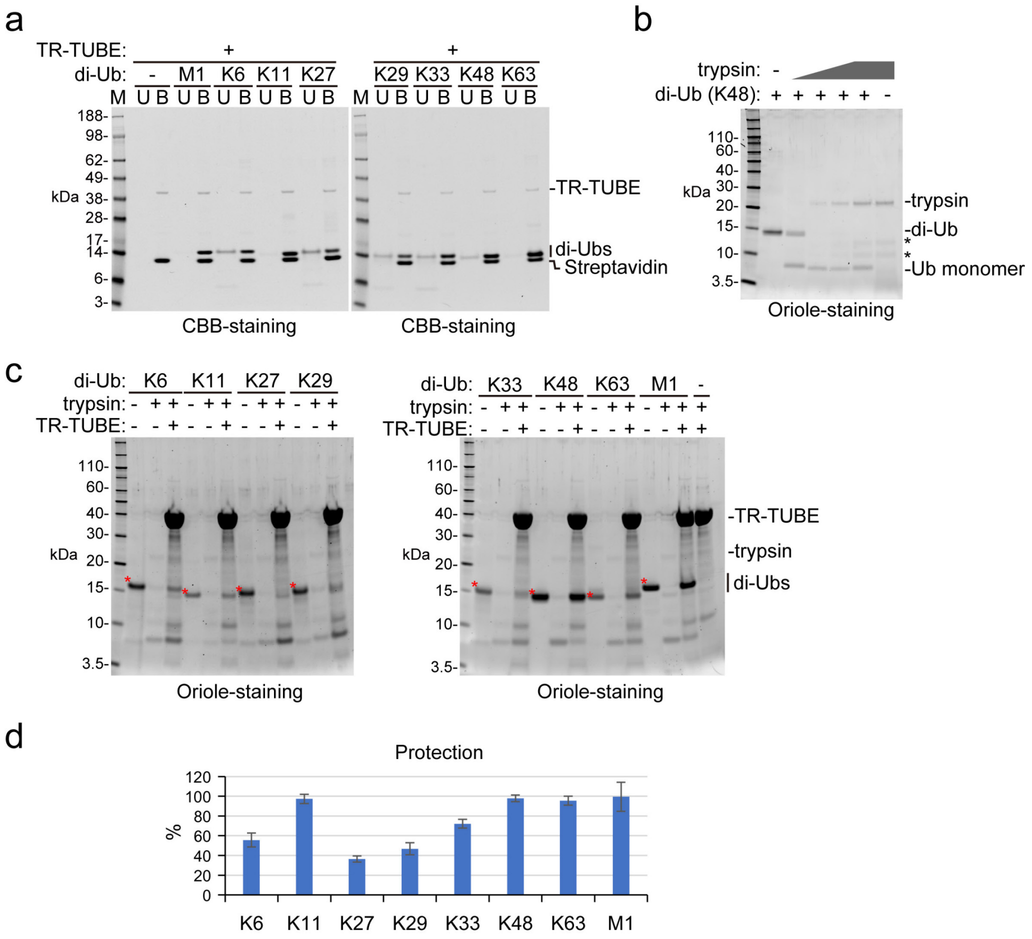

Supplementary Figure 2. Linkage-type selectivity of TR-TUBE and Ub-ProT assay of di-ubiquitins.

(*a*) Binding assay of TR-TUBE and di-ubiquitins. TR-TUBE (2 μg) was immobilized to streptavidin-conjugated Dynabeads, and then incubated for 30 min with eight different types of di-ubiquitins linked through K6, K11, K27, K29, K33, K48, K63, or M1 (2 μg). Unbound (U) and bound (B) proteins were analyzed by SDS-PAGE. The protein bands corresponding to TR-TUBE, di-Ubs, and streptavidin are indicated.

(*b*) Optimization of the amount of trypsin for complete cleavage of K48-linked di-ubiquitin. K48-linked di-ubiquitin (500 ng) was incubated overnight at 37°C with trypsin (25, 50, 100, or 200 ng). Asterisks indicate the degradation product of trypsin.

(*c*) Ub-ProT assay of di-ubiquitins. Di-ubiquitin (500 ng) was incubated overnight at 37°C with TR-TUBE (5 μg) and trypsin (50 ng). Di-ubiquitins are marked with red asterisks.

(*d*) Signal intensities of each di-Ubs, trypsinized sample, or TR-TUBE protection sample as in (*c*) were quantified and relative protection levels of each di-Ub are represented (means ± SEM of three independent experiments; Supplementary Data 1).

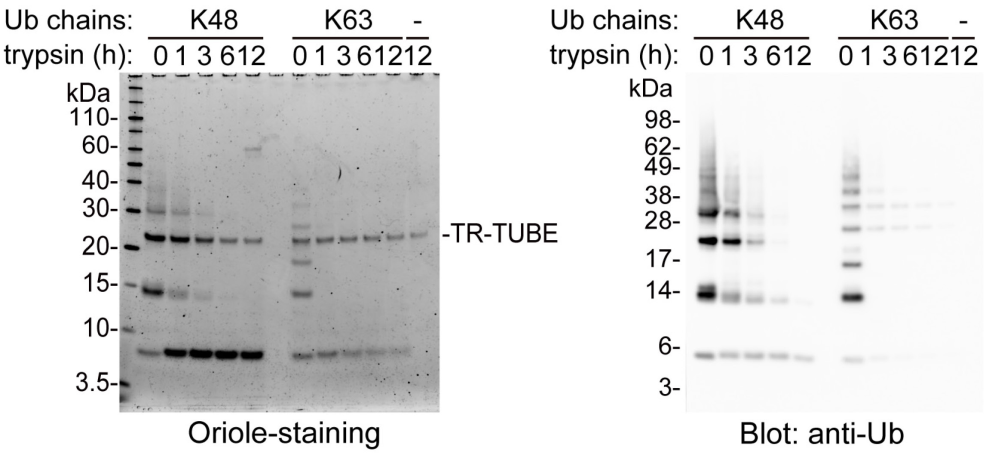

Supplementary Figure 3. Trypsinizaton of polyubiquitin chains.

Unanchored K48 or K63-linked polyubiquitin chains (500 ng) were incubated with trypsin (250 ng) at 37°C for the times indicated. The chains were visualized by Oriole staining (left) or analyzed by immunoblotting with anti-Ub antibody (P4D1; right).

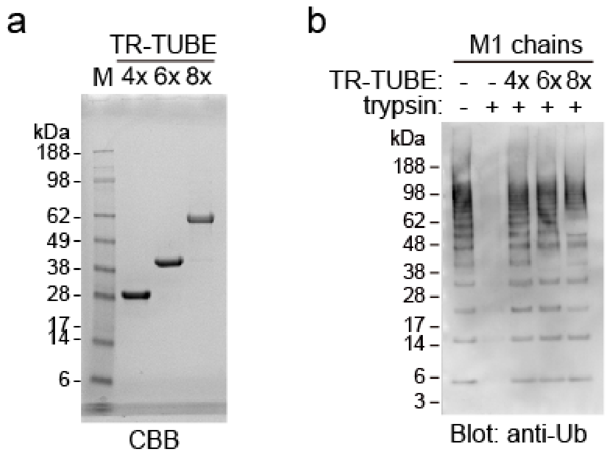

Supplementary Figure 4. Ub-ProT assay using TR-TUBEs with different numbers of UBA domains.

(*a*) Expression and purification of 4×TR-TUBE, 6×TR-TUBE, and 8×TR-TUBE. Purified proteins were analyzed by SDS-PAGE.

(*b*) M1-linked chains were subjected to Ub-ProT assay with 4×TR-TUBE, 6×TR-TUBE, and 8×TR-TUBE, followed by immunoblotting with anti-ubiquitin antibody.

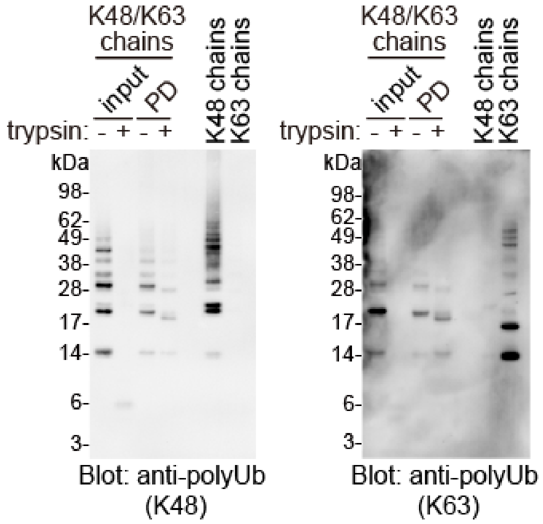

Supplementary Figure 5. Ub-ProT assay of K48/K63 branched chains.
K48/K63 branched chains were subjected to Ub-ProT assay with TR-TUBE, followed by immunoblotting with linkage specific antibodies (K48-linked: EP8589, K63-linked: EPR8590-448).

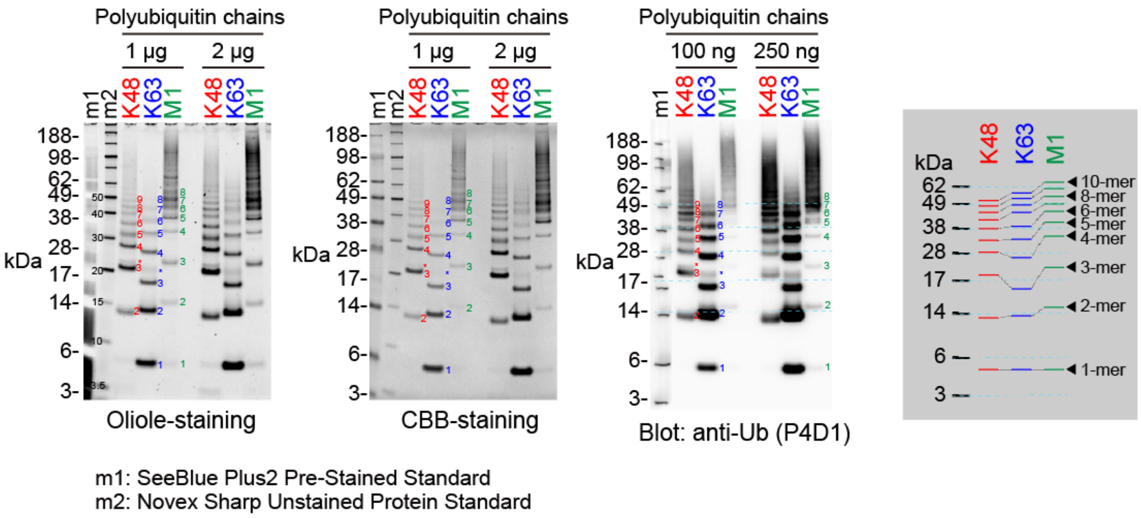

Supplementary Figure 6. Gel mobilities of K48-, K63-, and M1-linked polyubiquitin chains.

Different amounts of K48-, K63-, and M1-linked chains were electrophoresed on 4–12% NuPAGE gel with MES running buffer (Life Technologies). The chains were visualized by Oriole or CBB staining (left and left middle). The chains were also analyzed by immunoblotting with anti-Ub antibody (P4D1; right middle). Clearly separated chains were numbered by different colors. We used two different protein standards (SeeBlue Plus 2 and Novex Sharp Unstained; Life Technologies) and the relative positions in the gel are summarized at right.

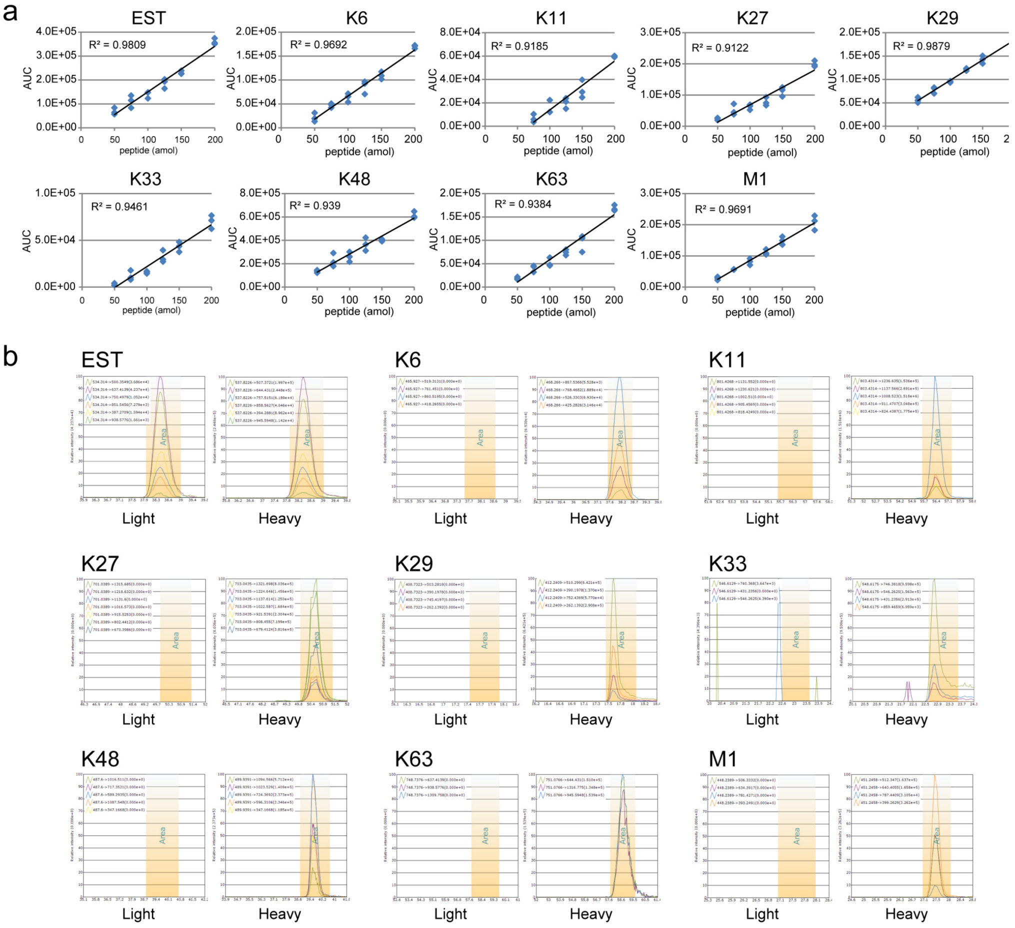

Supplementary Figure 7. Standard curves of Ub-AQUA/PRM and the effect of iodoacetamide treatment.

(*a*) Determination of the lower limit of quantitation (LLOQ) of Ub peptides. Ub peptides were spiked at concentrations of 50, 75, 100, 125, 150, or 200 amol in 500 ng of yeast matrix. The calculated standard curve regression line concentrations are presented. The acceptable range of variation was set by the FDA guidelines for the LLOQ (± 20%). Thus, the LLOQ of all peptides was determined to 50 amol.

(*b*) Effect of iodoacetamide treatment on ubiquitin quantitation. Previous studies have shown that iodoacetamide (IAA) treatment at high concentrations (55 mM) can lead to artifacts that chemically induce pseudo-ubiquitylation[2-4]. We used 10 mM IAA for preparation of our yeast lysates. To investigate whether IAA treatment affects ubiquitin quantitation, ubiquitin monomers were treated with 50 mM IAA for 30 min at room temperature and analyzed by ubiquitin PRM. Chromatograms of selected product ions of light (IAA-treated Ub) and heavy (internal AQUA) peptides are represented. Under this condition, pseudo-Ub peptides (light in each panel) were not detected, suggesting that pseudo-ubiquitylation was negligible in this study.

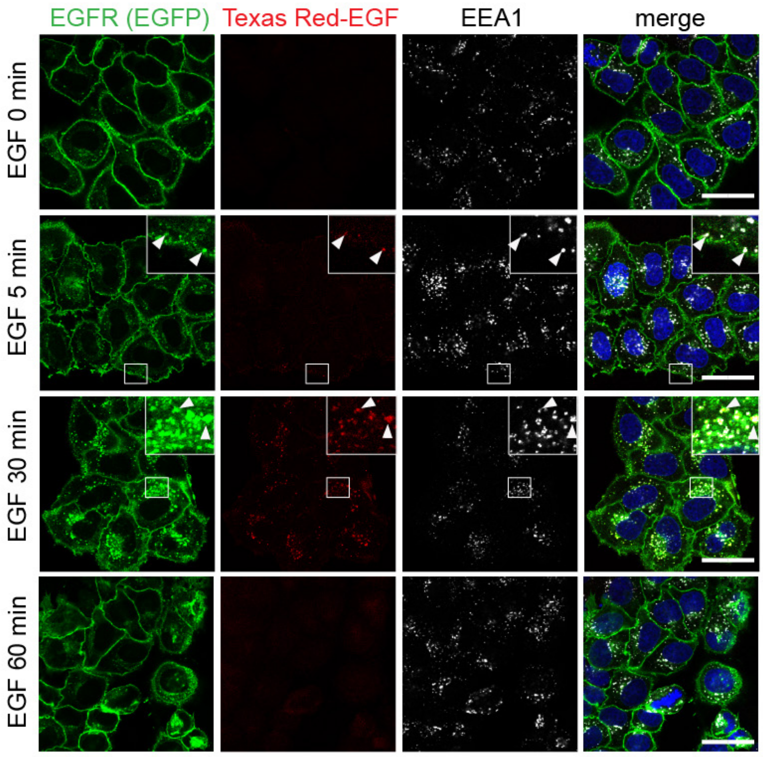

Supplementary Figure 8. Microscopic analysis of EGFR trafficking.

HeLa cells stably expressing EGFR-EGFP-3×FLAG were stimulated with 100 ng ml^{-1} of Texas red–conjugated EGF for the indicated times. Cells were fixed and stained with antibody against EEA1, an early endosome marker. Insets show higher magnification images of regions indicated by squares. Arrowheads indicate co-localization of EGF, EGFR, and Baf A1. Scale bars = 40 μm.

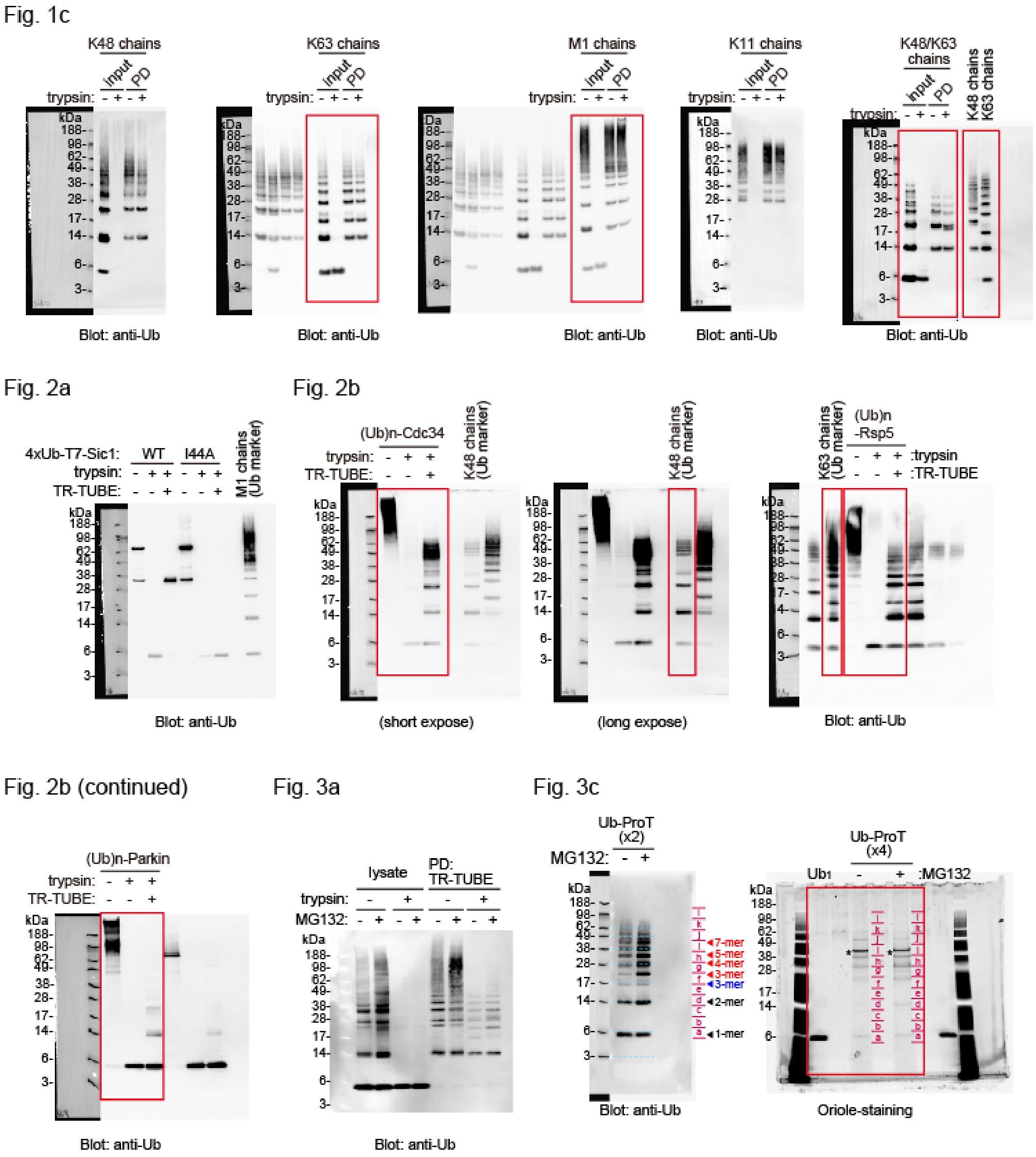

(continued)

Supplementary Figure 9. Uncropped blot and gel images in this study.

Fig. 4a

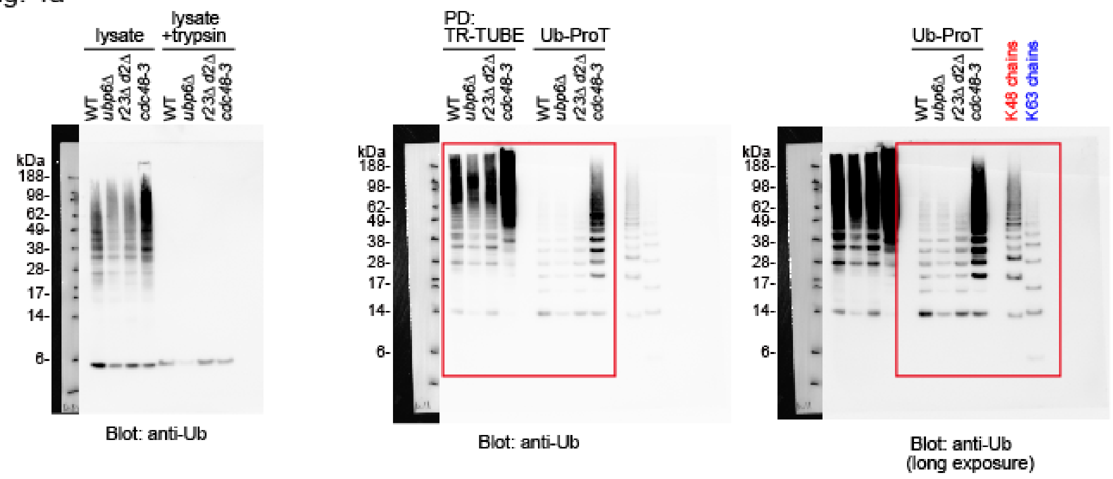

Fig. 5a

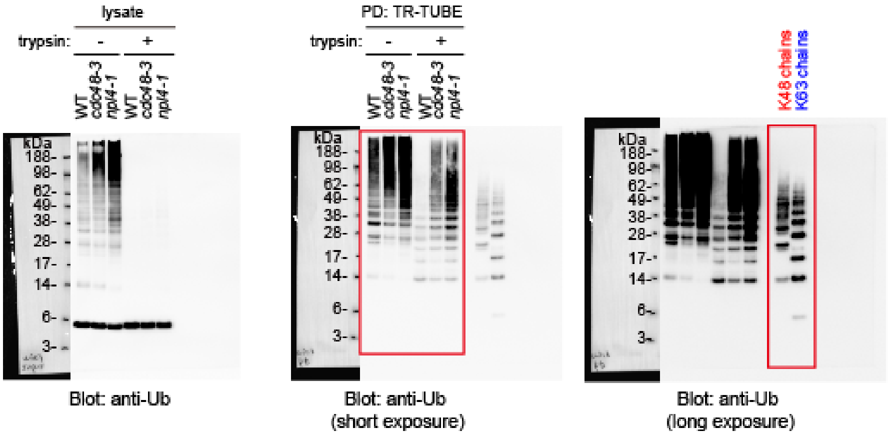

Fig. 5b

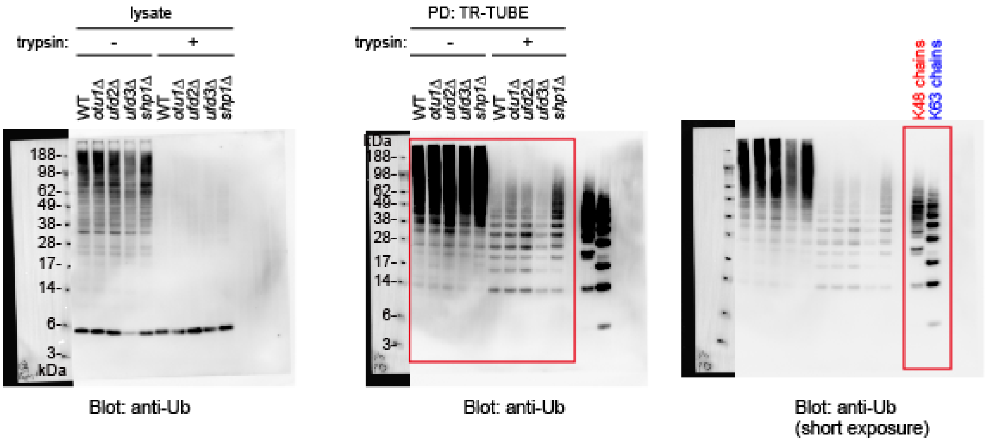

(continued)

Figure 6a

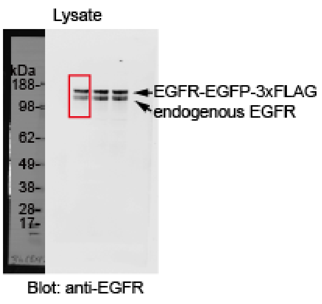

Figure 6b

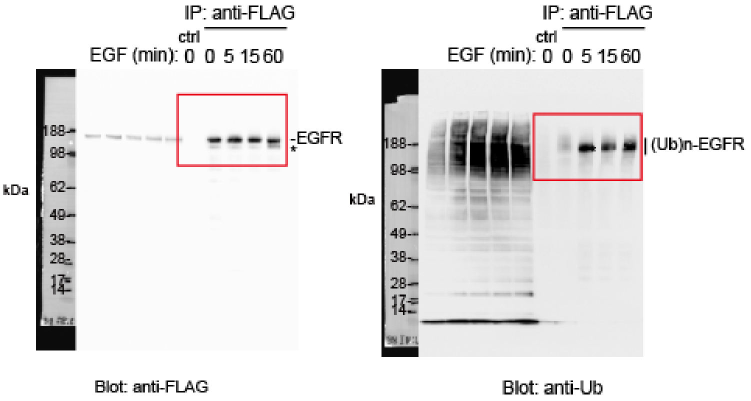

Fig. 6b

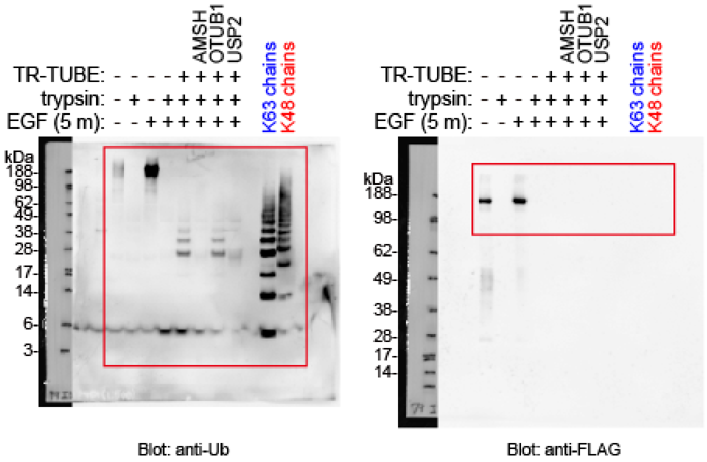

Fig. 6e

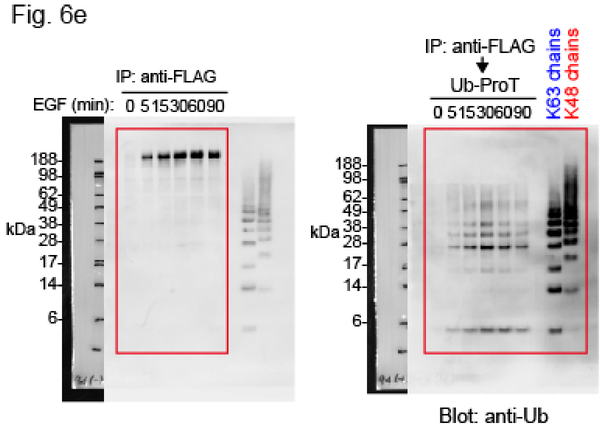

Supplementary Figure 9 continued

(*continued*)

Supplementary Figure 1c

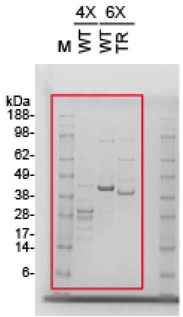

CBB-staining

Supplementary Figure 1d

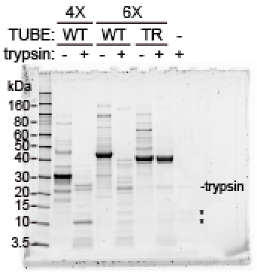

Oriole-staining

Supplementary Figure 2a

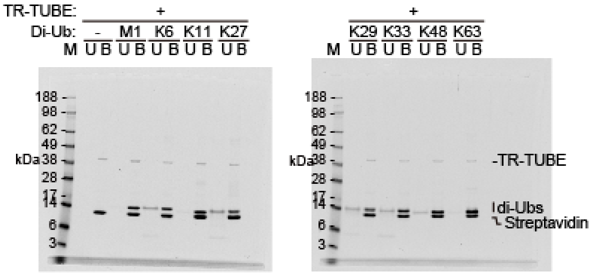

CBB-staining

CBB-staining

Supplementary Figure 2b

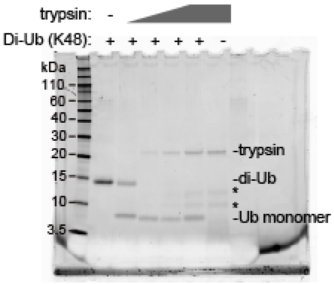

Oriole-staining

Supplementary Figure 2c

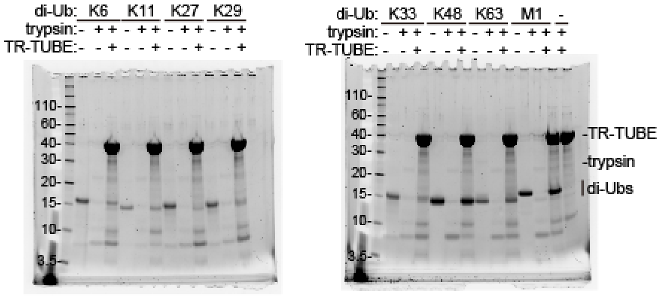

Oriole-staining

Oriole-staining

Supplementary Figure 3

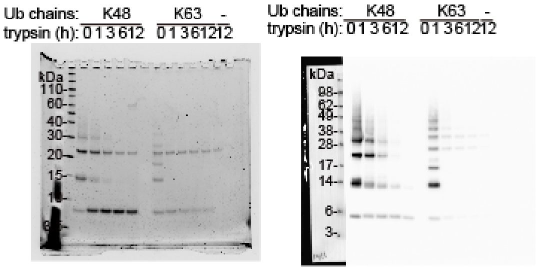

Oriole-staining

Blot: anti-Ub

Supplementary Figure 4

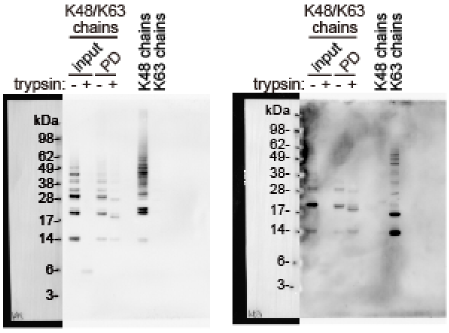

Blot: anti-polyUb (K48)

Blot: anti-polyUb (K63)

(*continued*)

Supplementary Figure 5a

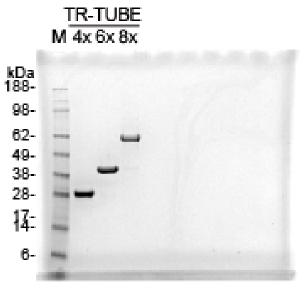

CBB-staining

Supplementary Figure 5b

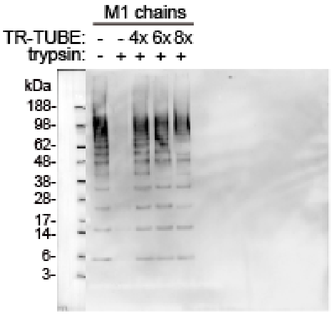

Blot: anti-Ub

Supplementary Figure 6

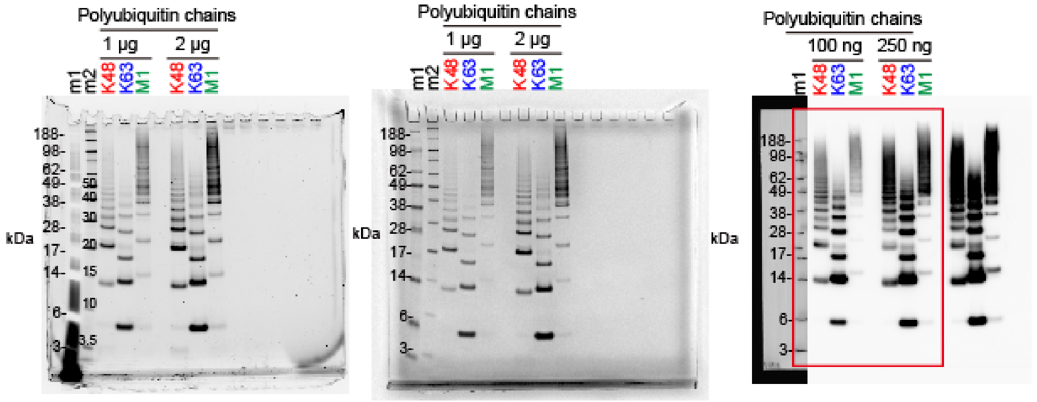

Oriole-staining

CBB-staining

Blot: anti-Ub (P4D1)

Supplementary Table 1. Yeast strains used in this study.

Strain name	Genotype	Reference
W303-1A	*MATa ura3-1 trp1-1 leu2-3,112 his3-11,15 ade2-1 can1-100*	Our stock
YYS1325	*MATa ura3-1 trp1-1 leu2-3,112 his3-11,15 ade2-1 can1-100 pdr5Δ:: HphMX lys2Δ::LEU2* (W303 background)	5
YHT38	*MATa ura3-1 trp1-1 leu2-3,112 his3-11,15 ade2-1 can1-100 ubp6Δ::KanMX pdr5Δ::HphMX lys2Δ::LEU2* (W303 background)	This study
YHT41	*MATa ura3-1 trp1-1 leu2-3,112 his3-11,15 ade2-1 can1-100 dsk2Δ::TRP1 rad23Δ::URA3 pdr5Δ::KanMX lys2Δ::LEU2* (W303 background)	This study
Y202	*MATα ura3-1 trp1-1 leu2-3,112 his3-11,15 ade2-1, can1-100 cdc48-3* (W303 background)	6
YYS2060	*MATα ura3-1 trp1-1 leu2-3,112 his3-11,15 ade2-1, can1-100 pdr5Δ::KanMX lys2Δ::LEU2 cdc48-3* (W303 background)	7
YHT235	*MATα ura3-1 trp1-1 leu2-3,112 his3-11,15 ade2-1, can1-100 npl4::npl4-1-KanMX* (W303 background)	This study
BY4741	*MATa his3Δ1 leu2Δ0 met15Δ0 ura3Δ0*	Open Biosystems
Y5665	*MATa his3Δ1 leu2Δ0 met15Δ0 ura3Δ0 otu1Δ::KanMX4* (BY4741 background)	Open Biosystems
Y3888	*MATa his3Δ1 leu2Δ0 met15Δ0 ura3Δ0 ufd2Δ::KanMX4* (BY4741 background)	Open Biosystems
Y5063	*MATa his3Δ1 leu2Δ0 met15Δ0 ura3Δ0 ufd3Δ::KanMX4* (BY4741 background)	Open Biosystems
Y3084	*MATa his3Δ1 leu2Δ0 met15Δ0 ura3Δ0 shp1Δ::KanMX4* (BY4741 background)	Open Biosystems

Supplementary References

1. Hjerpe, R. *et al.* Efficient protection and isolation of ubiquitylated proteins using tandem ubiquitin-binding entities. *EMBO reports* **10**, 1250-1258 (2009).
2. Nielsen, M.L. *et al.* Iodoacetamide-induced artifact mimics ubiquitination in mass spectrometry. *Nature methods* **5**, 459-460 (2008).
3. Xu, P. *et al.* Quantitative proteomics reveals the function of unconventional ubiquitin chains in proteasomal degradation. *Cell* **137**, 133-145 (2009).
4. Shi, Y., Xu, P. & Qin, J. Ubiquitinated proteome: ready for global? *Molecular & cellular proteomics : MCP* **10**, R110 006882 (2011).
5. Saeki, Y. *et al.* Lysine 63-linked polyubiquitin chain may serve as a targeting signal for the 26S proteasome. *EMBO J* **28**, 359-371 (2009).
6. Kimura, Y. *et al.* An inhibitor of a deubiquitinating enzyme regulates ubiquitin homeostasis. *Cell* **137**, 549-559 (2009).
7. Tsuchiya, H. *et al.* In vivo ubiquitin linkage-type analysis reveals that the Cdc48-Rad23/Dsk2 axis contributes to K48-linked chain specificity of the proteasome. *Molecular cell* **66**, 488-502.e487 (2017).
