# Supplementary material for: Ub-ProT reveals global length and composition of protein ubiquitylation in cells
Source: Nat Commun. 2018 Feb 6;9:524. doi: 10.1038/s41467-018-02869-x (PMC5802829; doi:10.1038/s41467-018-02869-x)
Supplement: Supplementary file 2 — Descriptions of Additional Supplementary Files [file 41467_2018_2869_MOESM2_ESM.pdf]

Descriptions of Additional Supplementary Files:

File Name: Supplementary Data 1
Description: (Dataset of Supplementary Fig. 2d)

File Name: Supplementary Data 2
Description: (Dataset of Fig. 3b)

File Name: Supplementary Data 3
Description: (Dataset of Fig. 3d)

File Name: Supplementary Data 4
Description: (Dataset of Supplementary Fig. 7a)

File Name: Supplementary Data 5
Description: (Dataset of Fig. 4b)

File Name: Supplementary Data 6
Description: (Dataset of Fig. 4c)

File Name: Supplementary Data 7
Description: (Dataset of Fig. 6c)
